# Chromoblastomycosis in India: Review of 169 cases

**DOI:** 10.1371/journal.pntd.0005534

**Published:** 2017-08-03

**Authors:** Reshu Agarwal, Gagandeep Singh, Arnab Ghosh, Kaushal Kumar Verma, Mragnayani Pandey, Immaculata Xess

**Affiliations:** 1 Department of Microbiology, All India Institute of Medical Sciences, Ansari Nagar, New Delhi, India; 2 Department of Dermatology & Venereology, All India Institute of Medical Sciences, Ansari Nagar, New Delhi, India; University of California San Diego School of Medicine, UNITED STATES

## Abstract

Chromoblastomycosis (CBM) is a chronic, progressive, cutaneous and subcutaneous fungal infection following the traumatic implantation of certain dematiaceous fungi. The disease has worldwide prevalence with predominant cases reported from humid tropical and subtropical regions of America, Asia, and Africa. Diagnosis is often delayed or misdirected either due to poor degree of clinical suspicions or clinical simulation of dermatological conditions. The infection is not uncommon in India and several case reports from the sub-Himalayan belt and western and eastern coasts of India have been published; however, very few have reviewed the cases. We reviewed 169 cases published in English literature from India during 1957 through May 2016, including 2 recent cases from our institute. A tremendous increase in the number of reported cases was noticed since 2012, since which, more than 50% of the cases had been published. A majority of the patients (74.1%) were involved in various agricultural activities directly or indirectly. The mean age at presentation was 43.3 years ± 16.0, with male to female ratio of 4.2:1. The duration of disease at the time of presentation varied from 20 days to 35 years. Any history of trauma was recalled only in 33.8% of the studied cases. The lower extremity was the most common site afflicted, followed by the upper extremity. The culture was positive in 80.3% of the cases with *Fonsecaea pedrosoi*, isolated as the most common fungal pathogen, followed by *Cladophialophora carrionii*. Although all the commercially available antifungals were prescribed in these cases, itraconazole and terbinafine were the most commonly used, either alone or in combination with other drugs/physical methods, with variable degrees of outcome. Combinations of different treatment modalities (chemotherapy and physical methods) yielded a cure rate of 86.3%. CBM is refractory to treatment and no single antifungal agent or regimen has demonstrated satisfactory results. Increased awareness with early clinical suspicion of the disease and adequate therapy are necessary to improve the outcome. However, depending upon the causative agent, disease severity, and the choice of antifungals, variable outcomes can be observed.

## Introduction

Chromoblastomycosis (CBM) is a chronic, progressive, cutaneous and subcutaneous fungal infection following the traumatic implantation of certain dematiaceous fungi through the skin of exposed body parts. The disease has worldwide prevalence with predominant cases reported from humid tropical and subtropical regions of America, Asia, and Africa [1]. The fungi causing CBM are ubiquitous, found in soil and decaying plant debris, including wood. As CBM is an implantation mycosis, occupation seems to play an important role [2]. The rural population, predominantly male, involved in various agricultural and outdoor activities are at a higher risk. A small, single, localized papule, nodule, plaque, or verrucous lesion is seen at the site of inoculation. Severe clinical forms and dissemination via lymphatics/hematogeneous/contiguous spread are rarely seen. The hallmark of CBM is “sclerotic bodies,” “medlar bodies,” or “muriform cells,” which can be demonstrated in potassium hydroxide (KOH) mount and hematoxylin and eosin staining [3]. *Fonsecaea pedrosoi*, *Phialophora verrucosa*, and *Cladophialophora carrionii* are the most frequent etiological agents of CBM. Less frequently reported are *Rhinocladiella aquaspersa* and *Exophiala dermatitidis*. Although mainly causing phaeohyphomycosis, *E*. *jeanselmei* and *E*. *spinifera* do produce muriform cells in CBM [4]. Recently, various other agents of CBM such as *F*. *monophora* [5], *F*. *nubica* [6], and *P*. *richardsiae* [7] have been reported. Diagnosis is often delayed or misdirected either due to poor degree of clinical suspicions or clinical simulation of dermatological conditions. Though known for 100 years, CBM still poses a therapeutic challenge to clinicians due to its recalcitrant nature and common relapse after treatment.

The infection is not uncommon in India and several case reports from the sub-Himalayan belt and western and eastern coasts of India have been published [8]. A very few case series describing the clinical manifestations, therapy, and outcome are available from India. We herein report 2 cases from our institute and review those reported in the Indian literature to provide the descriptive data on the epidemiology, clinical features, therapeutic regime practiced, and outcome of the disease.

### Case 1

A 27-year-old housewife, resident of Uttarakhand, presented to the dermatology department with complaint of progressive papulonodular lesions on the right leg for the last 8 years. Initially, she developed asymptomatic erythematous to brownish papules 5 x 5 mm in size on the lower part of the right leg, which later increased in number and size. No history of trauma or injury was recalled. She gave the history of prior treatment with terbinafine 250 mg BD for 6–7 months and itraconazle 100 mg BD for 1 year and 200 mg BD for 2 years. As the lesions remained static, she was referred to our tertiary care hospital in November 2014. On examination, multiple discrete, round, erythematous papules coalescing to form plaques were observed on the anterior aspect of lower third of the right leg. Each papule was 6 x 7 mm, firm, and nontender. The plaques were arranged in an annular pattern with central clearing. She was advised to continue itraconazole 200 mg BD for another 2 weeks. Failing to achieve clinical improvement in December 2014, the treatment modified to itraconazole 100 mg BD and ketoconazole 200 mg BD for 5.5 months. Initially, signs of clinical improvement were evident. There was reduction in erythema, lesions were flattened, and no new lesions developed. In May 2015, ketoconazole was stopped and itraconazole was increased to 200 mg BD. However, no evidence of improvement was further noticed. The rest of the history and physical examination were unremarkable. Routine haematological and biochemical investigations were within normal limits. The skin biopsy was taken from the lesion and sent for histopathological examination and fungal and mycobacterial culture. On histopathology, chronic inflammatory infiltrates comprising lymphocytes, plasma cells, and giant cells containing brown pigmented structures were seen. Microscopic examination of the biopsy specimen using 20% KOH showed small, round, thick-walled, brownish, septate sclerotic bodies measuring 6–12 μm. The specimen was cultured on Sabouraud dextrose agar (SDA) with antibiotics and incubated at 25°C and 37°C. Slowly growing colonies were observed at 25°C after 7–10 days. The colonies were jet black, velvety, and embedded in the medium with reverse black. Based on lactophenol cotton blue (LPCB) preparation and slide culture, the isolate was identified as *F*. *pedrosoi*. The identity of the isolate was confirmed by sequencing the internal transcribed spacer (ITS) region by using the ITS1 and ITS4 primers. Comparison of the nucleotide sequence of our isolate with the GenBank database using the BLAST algorithm yielded 99% homology with *F*. *pedrosoi* (accession number KX793110). The patient was started on liposomal amphotericin B (2 mg/kg x 18 days) and cryosurgery was done. The lesions regressed completely by the end of 11 months.

### Case 2

A 70-year-old farmer, a resident of Kumaon district in Uttarakhand, presented with a complaint of ulceroproliferative lesion on the dorsum of the right foot for 4 years. Following a minor trauma by a wooden stick, a small papule developed that progressed gradually to form a well-defined round plaque of size 1.5 x 2 cm. It was crusted and hyperkeratotic, with few black dots noted on the surface and oozing of seropurulent discharge. The surrounding area was edematous. The lesion progressively increased in size with superinfection due to *Staphylococcus aureus*, leading to the formation of a superficial abscess. Vitals were within normal limits. Haematological and biochemical parameters were normal. The patient was immunocompetent and nondiabetic.

Histopathological findings were suggestive of CBM. The microscopic examination in 20% KOH revealed the presence of sclerotic bodies. Culture at 25°C on SDA with antibiotics revealed small, smooth, folded, olive-black colonies after 10–12 days of incubation with reverse jet black. The fungus was identified as *Cladosporium* spp. on LPCB and slide culture. The identity of the isolate was confirmed by sequencing the ITS region using ITS1 and ITS4 primers. Comparison of the nucleotide sequence of our isolate with the GenBank database using the BLAST algorithm yielded 99% homology with *Cladosporium tenuissimum* (accession number KX793111). The patient was started on itraconazole 200 mg BD and showed marked reduction in the lesion after 7 months of treatment.

## Methods

### Literature search

The literature search was done in Medline (National Library of Medicine, Bethesda, Maryland, United States) and Google for the period of 1957 to May 2016 using the following terms: “chromoblastomycosis,” “chromomycosis,” “chromoblastomycosis dissemination,” “melanised fungi,” “dematiaceous fungi,” “*Fonsecaea*,” “*Phialophora*,” “*Cladophialophora*,” “*Exophiala*,” “*Hormodendrum*,” and “India.” Combinations such as “chromoblastomycosis in India,” “chromoblastomycosis and India,” “chromomycosis in India,” “chromomycosis and India,” “chromoblastomycosis dissemination in India,” and “chromomycosis dissemination and India” were used to retrieve the articles. Only cases from English literature were reviewed. Reference lists of retrieved articles were checked to detect additional articles missed by this search strategy. Cases of CBM, localized or disseminated, proven either by histopathology or in KOH mount, with or without culture proven, were included in the review.

### Data analysis

Data on demographic characteristics, occupation, geographical location, history of trauma, and clinical manifestations along with the duration, diagnostic modality, treatment given, and outcome were noted for each case. If the information on the resident state of the patient was unavailable, the institute from where the study had been reported was taken into consideration. Cases were considered as “immunocompromised” in which an underlying disease or predisposing factor was mentioned and as “immunocompetent” in which no such predisposing condition and negative HIV serology was mentioned. The data is presented as frequencies and percentages or mean and standard deviation (SD).

## Results

### Studies and cases

**Fig 1:**
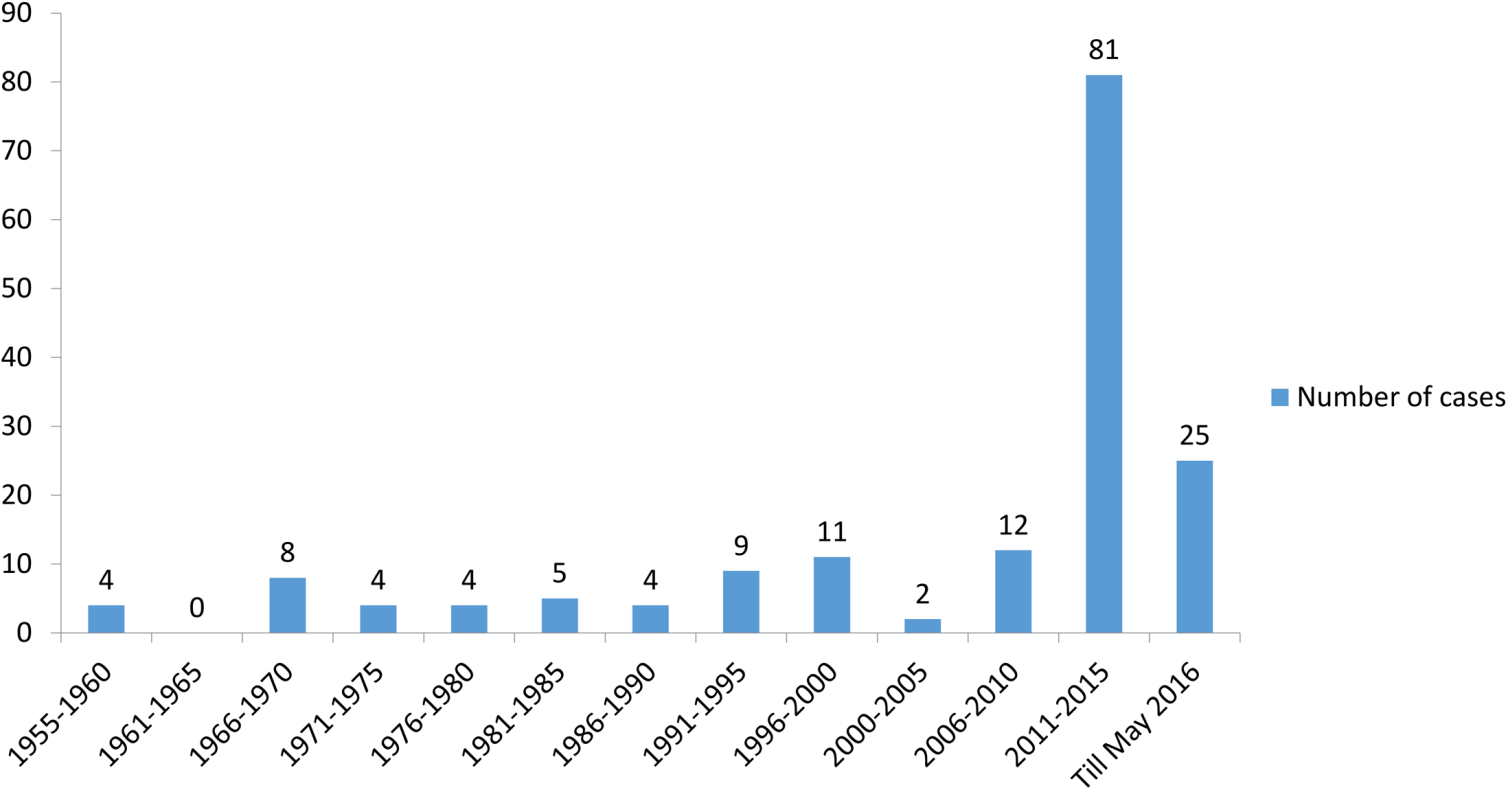
A time line of the number of cases of chromoblastomycosis reported from India since 1957.

Our literature search for the period of 1957 to May 2016 yielded 88 studies reporting 189 cases. Among these, 1 study was excluded so as to avoid duplication [9]. A case reported by Deshpande et al. with submandibular discharging sinus lacked sufficient evidence for the diagnosis of CBM and was excluded [10]. One patient whose 28-year follow-up was published in 2 different studies was counted as only a single case [11, 12]. A total of 167 cases were identified in 87 included studies. In addition to these, 2 recent cases from our institute were also included. Hence, a total of 169 cases were evaluated. There has been a tremendous increase in the number of cases reported after 2011 (Fig 1).

### Age and gender

**Fig 2:**
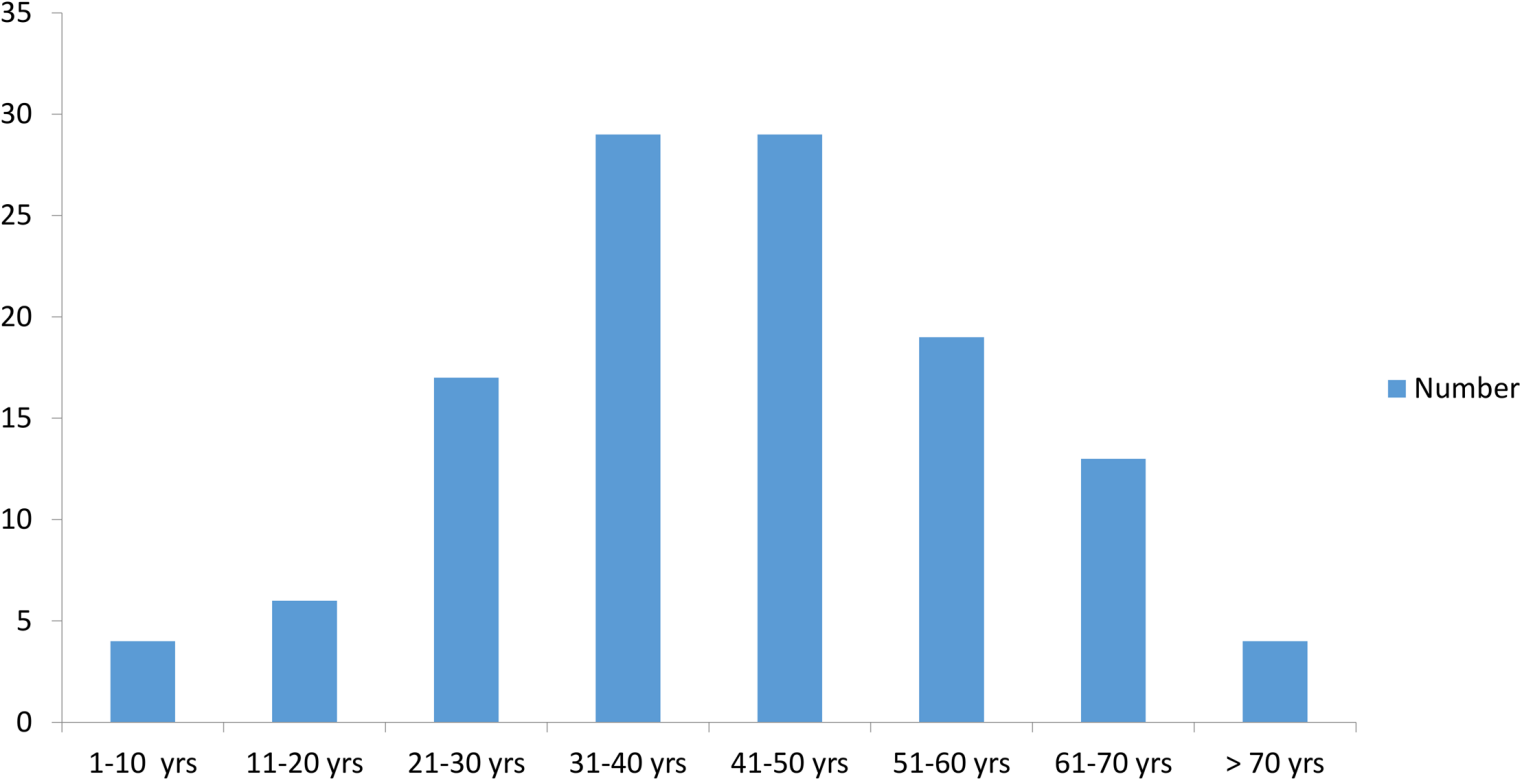
The figure shows age-wise distribution of cases with chromoblastomycosis in India.

The mean age at presentation was 43.3 years ± 16.0 (range 7–81 years old, data available for 121/169 cases). The information regarding the gender was available for 158 patients, out of whom, 128 (81%) were males and 30 (18.9%) were females. The male to female ratio was 4.2:1. The mean age of presentation among male patients was 44.1 years ± 16.2 (range 7–81 years old, data available for 96/121 cases) and female was 40.2 years ± 14.5 (range 10–67 years old, data available for 25/121 cases) (Fig 2).

### Geographical location

**Fig 3:**
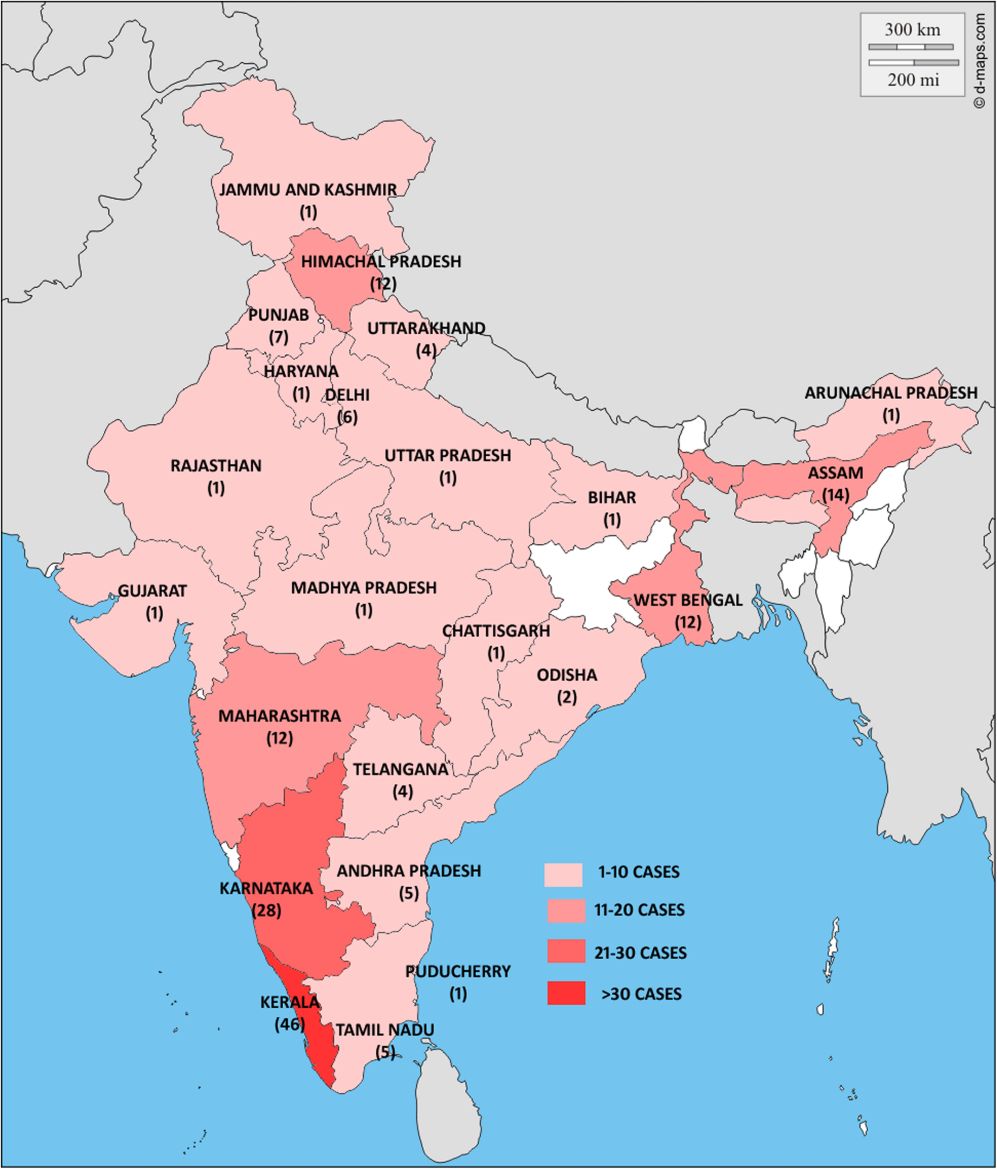
The map of India is depicted here with the state-wise distribution of cases.

The data on geographical distribution was available for 168 cases. The highest number of cases had been reported from Kerala (46 cases) followed by Karnataka (28 cases), Assam (14 cases), Himachal Pradesh, and Maharashtra (12 cases each) (Fig 3).

### Occupation

The occupational history was available for 112 cases. A majority of the patients (74.1%) were either farmers or involved in various agricultural activities directly or indirectly, followed by manual labourers (7.1%).

### History of trauma

A majority of the studied patients (66.1%) either could not recall or gave a negative history of trauma prior to their manifestations. A history of trauma was positive in 33.8% of the studied cases (data available for 119/169 cases).

### Underlying condition or predisposing factors

Of 169 cases, 10 (5.9%) had a history of underlying immunosuppression. The underlying conditions were renal transplant, gall bladder carcinoma, skin allograft, HIV, and leprosy. History of corticosteroids intake was documented in 9 cases.

### Duration of disease

The duration of disease at the time of presentation varied from 20 days to 35 years (mean 5.76 years, data available for 114/169 cases).

### Site of involvement

The lesions were localized to 1 site in 90.3% of cases (150/166, data available for 166 cases). The most common site afflicted was lower limb (96 cases) followed by upper limb (33 cases), face (12 cases), and trunk (6 cases). In 3 cases, unusual sites alone such as genitals and axilla were involved. More than 1 site was affected in 9.6% (16/166) of cases. Extracutaneous spread involving regional lymph nodes, pleural cavity, oropharynx, trachea, larynx, and ileocecal regions was seen in 8 cases. Distant bone lesions were reported in 3 cases.

### Type of the lesion

The most common clinical variety described was plaque (54.7%) followed by verrucous (23.9%), nodular (6.8%), tumoral, and ulcerative (4.7% each). More than 1 type of lesion was observed in 4.7% of the cases (data available for 146/169 cases).

### Mycological culture

The causative organism was isolated in 80.3% of the cases (127/158, data available for 158 cases). The cultures were either negative or not done in 13.2% and 5.0% of cases, respectively. The contaminants were grown in 1.2% (2 cases). Among the culture positives, *Fonsecaea* spp. was the most common etiological agent (66.1%) followed by *Cladophialophora* spp. (25.1%) and *Phialophora* spp. (3.9%). Others include *Bipolaris* spp. (2 cases), *Exophiala* spp. (2 cases), *Curvularia lunata* (1 case), and *Rhytidhysteron* spp. (1 case). The 2 most common fungi isolated were *F*. *pedrosoi* (66 cases) followed by *C*. *carrionii* (20 cases) (S1 Table).

### Therapeutic regimen

The details of different treatment modalities were described for 103 cases (Table 1). The most commonly prescribed drug, either alone or in combination with other drugs/physical methods, was itraconazole in 55.5% followed by terbinafine in 22.3%, potassium iodide in 18.4%, fluconazole in 13.5%, amphotericin B in 7.7%, and ketoconazole in 7.7% of the studied subjects. Less commonly used were hamycin and antihistamines (3 cases), nystatin (1 case), 5 flurouracil (2 cases), 5 fluorocytosine (1 case), griseofulvin (2 cases), isonicotinoic acid hydrazide (1 case), and thiabendazole/mebendazole (4 cases). Other treatment modalities such as surgical excision alone and cryotherapy alone were done in 5 and 2 cases, respectively.

**Table 1:** The table shows the outcome of various treatment modalities.

Outcome	Chemotherapy only (*n* = 74)	Surgical excision only (*n* = 5)	Cryotherapy only (*n* = 2)	Chemotherapy + surgical excision (*n* = 9)	Chemotherapy + cryotherapy/CO_2_ laser/ superficial X- ray (*n* = 13)
Cured	56	4	2	7	12
Partial cured	5	-	-	-	1
Not cured	2	1*	-	1*	-
Lost to follow-up	6	-	-	1	-
Died	2	-	-	-	-
Data not available	3	-	-	-	-

*amputation done

- non-applicable

### Outcome

The documentation of clinical outcome of the disease was available for 102 cases. The outcome was described as cure in 82, partial cure in 6, and not cured in 2 cases. The subjects were lost to follow-up in 8 cases and mortality was seen in 2 cases. Amputation was performed in 2 cases.

## Discussion

This article gives an overview on epidemiology, clinical manifestations, treatment, and outcome of CBM in our country. Originally reported from Brazil in 1914 [13], the first case from India was reported in 1957 [14]. Since then, a large number of cases have been reported from India. In the last review from India in 1999, 34 cases were included [15]. A tremendous increase in the number of reported cases in the last 4–5 years was noticed, a period in which more than 50% of the cases had been published (68 cases were published between 1957–2011 as compared to 101 cases from 2012 to May 2016). This might be due to the increased awareness among clinicians to suspect or diagnose the disease, better laboratory support, or increased awareness to publish the cases.

In our study, a large number of cases had been reported from the western and eastern coasts, southern India, northeastern areas, and the sub-Himalayan belt, namely Karnataka, Kerala, Maharashtra, West Bengal, Assam, and Himachal Pradesh, due to the hot and humid climate, which is essential for fungi to thrive. In addition to the climate, dense forest area with plenty of vegetation and agriculture as an occupation further predispose the natives to the disease. This is in concordance with what was reported in the previous reviews from India [8, 15]. A few sporadic cases had also been reported from nonendemic areas such as Bihar, Chattisgarh, Jammu and Kashmir, Rajasthan, and Uttar Pradesh, highlighting the importance of keeping CBM in differentials for cutaneous lesions, irrespective of the geographical distribution.

The disease is more prevalent in males 30–50 years of age, with a male to female ratio varying from 5:1to 9:1 in various studies [16–19]. Infections in children and adolescents have also been reported [20, 21]. Our study also showed the same pattern, with males outnumbering females, the latter accounting for 18.9%. More than 50% of affected cases were in the age group of 31–60 years. This is due to the fact that in Indian settings, males (the sole earning members of their families), are more commonly involved in outdoor activities, exposing them to the causative agents. In countries like Brazil and Japan, where women are exposed to same working conditions as men, the prevalence of infection is almost same [22, 23]. Adolescents and children were involved in 5.7% cases.

The infection usually follows the inoculation of soil or vegetative matter contaminated by dematiaceous fungi or via penetration of foreign bodies such as wood splinters or thorns or minor trauma or abrasions [2]. In the present study, history of any trauma was recalled only in 33.8% of cases. The absence of trauma in a majority of cases was attributed to the fact that either it went unnoticed or was not recalled, as the symptoms do not appear for years.

The fungus causing the disease lives in soil and due to work-related trauma, infection occurs most frequently among the barefoot farmers or those involved in various agricultural activities. More than 70% of those affected in our study were involved in agricultural work and were of rural background. The other risk factors were poor nutrition and hygiene habits.

The disease is usually gradual and indolent in nature. Therefore, patients often seek medical advice years after acquiring infection or developing skin lesions. Most of the studied cases were chronic, with a mean duration of 5.7 years. The longest duration was 35 years and the shortest was 20 days. Though the latter is unusual, there are some reports of fast progression of the disease, especially in the immunocompromised [24]. The lesions usually develop at the site of inoculation and were found chiefly in lower extremities in our study, followed by upper, which is similar to those reported in most regions of the world [23, 25]. This reinforces the fact that exposed parts of both extremities are commonly exposed to etiological agents, thus supporting the traumatic implantation theory. However, an exception to this is reported in a study of 290 cases from Japan, where CBM occurs more commonly on upper extremities in males and face or neck in females [26]. The trunk was the most common site afflicted in a study from Venezuela [15].

Lesions usually start as solitary, unilateral papules which may slowly progress to verrucous nodules and plaques and may ulcerate [27]. More than 1 type of lesion can develop in advanced, severe cases. Morphologically nodular is the most frequent variety followed by verrucous and plaquelike lesions [25, 28]. In contrast to this, plaque was the most common in our study, followed by verrucous, with 4.7% of cases showing mixed types of lesions.

Extracutaneous spread is believed to be low in CBM. The known sites of metastasis are the lymph nodes, brain, and lung [29]. However, the disease seldom involves bone [30]. The spread to regional lymph nodes, tonsillar regions, pleural cavity, trachea, larynx, and ileocecal region was reported in 7 cases [11, 14, 15, 31–34]. Fungus was demonstrated in all these organs either by histopathology or culture or both. The respiratory pathology was also found in another case; however, a fungus, *Geotrichum candidum*, different than that of cutaneous lesion was isolated [35]. Bone lesions were reported in 3 cases, among which, in 2 cases, lesions were not established to be directly due to fungi [34, 36]. An osteolytic lesion at the head of the metatarsal underlying the cutaneous lesion was considered to be due to contiguous spread in the third case [37].

Diagnosis is often obscured due to clinical simulation of various dermatological conditions such as cutaneous tuberculosis, leprosy, leshmaniasis, sporotrichosis, mycetoma, psoriasis, and malignancies such as verrucous carcinoma and cutaneous lymphoma [1]. Approximately 10% of the reviewed cases were misdiagnosed as cutaneous tuberculosis and were prescribed antitubercular treatment. Other masquerading or mimicking conditions included leprosy (2 cases), cutaneous leishmaniasis (2 cases), psoriasis, facial wart, sporotrichosis, and squamous cell carcinoma (1 case each).

Secondary infections, lymphedema, dissemination, and even squamous cell carcinoma in long-standing cases are possible sequelae [25, 38, 39]. Complications like secondary infection, genital elephantiasis, and squamous cell carcinoma were recorded in 3 cases [31, 40]. Though rare, dissemination either by contiguous spread via lymphatics or hematogenous route, or autoinoculation had also been reported [41–43]. In our study, dissemination via hematogenous or lymphatic route was noted in 5 cases [11, 15, 32, 44, 45] and 3 cases, respectively [46–48]. Both the routes were involved in a single case [34]. Recently, CBM has been increasingly reported in immunocompromised individuals. Renal transplant is one of the common underlying conditions. So far, 15 cases of CBM in renal transplant recipients have been described in the literature including 3 from India [49–57]. Other predisposing conditions reported from India included 1 case each of gall bladder carcinoma, skin graft, and HIV infection. CBM developed in 2 leprosy patients who were on long-term steroids. Following intralesional/oral corticosteroids, the disease was reported in another 2 cases. It had been observed that in such patients, partial suppression of cell-mediated immune response to fungus occurs at some point during infection and might have predisposed them to CBM [58]. These facts highlight the importance of keeping in mind the possibility of CBM in immunocompromised cases.

Another striking feature noted in the study was a case harboring concomitant mycetoma and CBM. To the best of our knowledge, to date, concurrent presence had been reported in 3 cases worldwide [59–61]. This might be due to sharing common climatic conditions for the survival and mode of transmission.

*F*. *pedrosi* and *C*. *carrionii* were the 2 most common fungal isolates in our study. Similar results are shown from other countries like Mexico, Cuba, the Dominican Republic, Venezuela, Australia, and South Africa [62–65]. Of these, *F*. *pedrosi* is common in humid tropical climates and *C*. *carrionii* is thought to be endemic in semiarid zones [30]. *C*. *carrionii* is the most common pathogen in Australia and Africa and *F*. *pedrosoi* in Venezuela, Cuba, and Japan [15]. However, in the present study, no particular distribution pattern was appreciated for any of the causative agents.

Due to the diversity in the etiological agents, bioavailablity of the antifungals, and variation in clinical manifestations, no single uniform treatment recommendations have been established to date. CBM is often difficult to treat and its refractoriness to various treatment modalities further complicates the situation. Depending upon the causative agents, severity of lesions, and choice of therapy, a variable cure rate of 15%–80%, with *F*. *pedrosoi* being less sensitive to antifungal therapy, had been reported [66]. The different therapeutic modalities advocated in the studied cases were chemotherapy and physical methods (cryosurgery/CO_2_ laser/superficial X-rays and surgical excision), either alone or in combination. Cure rates of 75.6% and 85.7% were achieved in cases treated alone either with chemotherapy or physical methods, respectively. However, the cure rate increased to 86.36% when the above 2 modalities were used in combination. The literature had shown the best results with oral terbinafine and itraconazole when used in high doses for long periods, and their combination was considered to be synergistic [67, 68]. Cure rates of 76.92% and 100%, respectively, were achieved when itraconazole and terbinafine alone were prescribed, and their combination yielded 100% success rate in our study. Failure to achieve any cure led to amputation in 2 cases.

## Supporting information

S1 Table Demographic and clinical details of all 169 cases included in the present study. (DOCX)

## References

[1] Yap FB. Chromoblastomycosis. Int J Infect Dis. 2010;14(6):e543–4. Epub 2009/11/06. doi: 10.1016/j.ijid.2009.07.005 . 19889559 10.1016/j.ijid.2009.07.005

[2] Queiroz-Telles F. Chromoblastomycosis: A Neglected Tropical Disease. Rev Inst Med Trop Sao Paulo. 2015;57 Suppl 19:46–50. Epub 2015/10/16. doi: 10.1590/S0036-46652015000700009 . 26465369 10.1590/S0036-46652015000700009 PMC4711190

[3] Ameen M. Chromoblastomycosis: clinical presentation and management. Clin Exp Dermatol. 2009;34(8):849–54. Epub 2009/07/07. doi: 10.1111/j.1365-2230.2009.03415.x . 19575735 10.1111/j.1365-2230.2009.03415.x

[4] Krzysciak PM, Pindycka-Piaszczynska M, Piaszczynski M. Chromoblastomycosis. Postepy Dermatol Alergol. 2014;31(5):310–21. Epub 2014/11/15. doi: 10.5114/pdia.2014.40949 . 25395928 10.5114/pdia.2014.40949 PMC4221348

[5] Najafzadeh MJ, Rezusta A, Cameo MI, Zubiri ML, Yus MC, Badali H, et al Successful treatment of chromoblastomycosis of 36 years duration caused by *Fonsecaea monophora*. Med Mycol. 2010;48(2):390–3. Epub 2009/06/03. doi: 10.1080/13693780903008813 . 19488919 10.1080/13693780903008813

[6] Najafzadeh MJ, Sun J, Vicente V, Xi L, van den Ende AH, de Hoog GS. *Fonsecaea nubica* sp. nov, a new agent of human chromoblastomycosis revealed using molecular data. Med Mycol. 2010;48(6):800–6. Epub 2010/03/23. doi: 10.3109/13693780903503081 . 20302550 10.3109/13693780903503081

[7] Son YM, Kang HK, Na SY, Lee HY, Baek JO, Lee JR, et al Chromoblastomycosis Caused by *Phialophora richardsiae*. Ann Dermatol. 2010;22(3):362–6. Epub 2010/08/17. doi: 10.5021/ad.2010.22.3.362 . 20711281 10.5021/ad.2010.22.3.362 PMC2917698

[8] Rajendran C, Ramesh V, Misra RS, Kandhari S, Upreti HB, Datta KK. Chromoblastomycosis in India. Int J Dermatol. 1997;36(1):29–33. Epub 1997/01/01. . 9071611 10.1046/j.1365-4362.1997.00008.x

[9] Bhat RM, Ramayivadakayil A, Monteiro R, Sukumar D, Srinath MK. A retrospective study of chromomycosis in a tertiary care institution in South India. Egyptian J Dermatol Venerol. 2014;34(2):126–9.

[10] Deshpande S, Sahni S, Murti P. Case reports of chromomycosis. Indian J Pathol Microbiol. 1993;36(4):469–73. Epub 1993/10/01. . 8157318

[11] Bharti R, Malhotra SK, Bal MS, Sharma K. Chromoblastomycosis. Indian J Dermatol Venereol Leprol. 1995;61(1):54–5. Epub 1995/01/01. . 20952879

[12] Kumar K, Sarin RC. Chromoblastomycosis Ind J Dermatol Venereol. 1978;44(5):310–5.

[13] Rudolph M. Über die brasilianische "Figueira" (Vorläufige Mitteilung). Arch Schiffs Tropen-Hyg. 1914;18:498–9.

[14] Kakoti LM, Dey NC. Chromoblastomycosis in India. J Indian Med Assoc. 1957;28(8):351–5. Epub 1957/04/16. . 13429115

[15] Sharma NL, Sharma RC, Grover PS, Gupta ML, Sharma AK, Mahajan VK. Chromoblastomycosis in India. Int J Dermatol. 1999;38(11):846–51. Epub 1999/12/03. . 10583618 10.1046/j.1365-4362.1999.00820.x

[16] Al-Doory Y. Chromomycosis In: AF DS, editor. Occupational mycoses. Philadelphia: Lea & Febiger; 1983 p. 95–121.

[17] Bonifaz A, Carrasco-Gerard E, Saul A. Chromoblastomycosis: clinical and mycologic experience of 51 cases. Mycoses. 2001;44(1–2):1–7. Epub 2001/06/12. . 11398635 10.1046/j.1439-0507.2001.00613.x

[18] Queiroz-Telles F, Esterre P, Perez-Blanco M, Vitale RG, Salgado CG, Bonifaz A. Chromoblastomycosis: an overview of clinical manifestations, diagnosis and treatment. Med Mycol. 2009;47(1):3–15. Epub 2008/12/17. doi: 10.1080/13693780802538001 . 19085206 10.1080/13693780802538001

[19] Queiroz-Telles F, Nucci M, Colombo AL, Tobon A, Restrepo A. Mycoses of implantation in Latin America: an overview of epidemiology, clinical manifestations, diagnosis and treatment. Med Mycol. 2011;49(3):225–36. Epub 2010/12/07. doi: 10.3109/13693786.2010.539631 . 21128710 10.3109/13693786.2010.539631

[20] Khan I, Khan AR, Khan MS. Clinicopathological study of cutaneous chromoblastomycosis in Pakistan. J Pak Assoc Dermatol. 2012;22:122–5.

[21] Perez-Blanco M, HernÃ¡ndez Valles R, Garcia-Humbria L, Yegres F. Chromoblastomycosis in children and adolescents in the endemic area of the Falcon State, Venezuela. Med Mycology. 2006;44(5):467–71. 10.1080/13693780500543238 16882614

[22] Kondo M, Hiruma M, Nishioka Y, Mayuzumi N, Mochida K, Ikeda S, et al A case of chromomycosis caused by *Fonsecaea pedrosoi* and a review of reported cases of dematiaceous fungal infection in Japan. Mycoses. 2005;48(3):221–5. Epub 2005/04/22. doi: 10.1111/j.1439-0507.2005.01089.x . 15842342 10.1111/j.1439-0507.2005.01089.x

[23] Silva JP, de Souza W, Rozental S. Chromoblastomycosis: a retrospective study of 325 cases on Amazonic Region (Brazil). Mycopathologia. 1998;143(3):171–5. Epub 1999/06/03. . 10353215 10.1023/a:1006957415346

[24] Sauerteig E, Hernandez R, Salfelder K, Bastidas C. Acute chromoblastomycosis provoked by an insect bite in an immunosuppressed patient. Mycoses. 1998;41(5–6):191–4. Epub 1998/08/26. . 9715631 10.1111/j.1439-0507.1998.tb00322.x

[25] Minotto R, Bernardi CD, Mallmann LF, Edelweiss MI, Scroferneker ML. Chromoblastomycosis: a review of 100 cases in the state of Rio Grande do Sul, Brazil. J Am Acad Dermatol. 2001;44(4):585–92. Epub 2001/03/22. doi: 10.1067/mjd.2001.112220 . 11260530 10.1067/mjd.2001.112220

[26] Fukushiro R. Chromomycosis in Japan. Int J Dermatol. 1983;22(4):221–9. Epub 1983/05/01. . 6345416 10.1111/j.1365-4362.1983.tb03371.x

[27] Lupi O, Tyring SK, McGinnis MR. Tropical dermatology: fungal tropical diseases. J Am Acad Dermatol. 2005;53(6):931–51, quiz 52–4. Epub 2005/11/29. doi: 10.1016/j.jaad.2004.10.883 . 16310053 10.1016/j.jaad.2004.10.883

[28] d'Avila SC, Pagliari C, Duarte MI. The cell-mediated immune reaction in the cutaneous lesion of chromoblastomycosis and their correlation with different clinical forms of the disease. Mycopathologia. 2003;156(2):51–60. Epub 2003/05/08. . 12733624 10.1023/a:1022948329193

[29] Rippon JW. Chromoblastomycosis Medical Mycology: The Pathogenic Fungi and the Pathogenic Actinomycetes. Philadelphia: W.B. Saunders; 1988 p. 276.

[30] Kwon-Chung KJ, Bennett JE. Chromoblastomycosis Medical Mycology. Pennsylvania: Lea and Febiger; 1992 p. 337.

[31] Jacob M, Mathai R, Prasad PVS, Bhaktaviziam A. Chromoblastomycosis with squamous cell carcinoma. Ind J Dermatol Venereol Leprol. 1988;54(6):314–7. 28134189

[32] Pavithran K. Disseminated chromoblastomycosis. Ind J Dermatol Venereol Leprol. 1991;57(3):155–6.

[33] Radhakrishnamurthy K. Chromomycosis due to *Phialophora pedrosoi*. Indian J Dermatol Venereol Leprol. 1981;47(5):281–4. 28211410

[34] Verma KC, Chaudhary SD, Chugh TD, Bhargava N. Chromoblastomycosis (A case report). Ind J Dermatol Venereol. 1977;43(1):35–7. 28266362

[35] Sharma NL, Sharma RC, Gupta ML, Singh P, Arora VK. Cutaneous chromomycosis with pulmonary geotrichosis. Ind J Dermatol Venereol Leprol. 1989;55(5):331–3. 28128150

[36] Battu V, Ramam M, Pasricha JS, Mohapatra LN. Chromomycosis with some unusual features. Ind J Dermatol Venereol Leprol. 1986;52(4):181–3. 28150606

[37] Sharma NL, Sharma VC, Mahajan V, Shanker V, Sarin S. Chromoblastomycosis with underlying osteolytic lesion. Mycoses. 2007;50(6):517–9. Epub 2007/10/20. doi: 10.1111/j.1439-0507.2007.01398.x . 17944717 10.1111/j.1439-0507.2007.01398.x

[38] Azevedo CM, Marques SG, Santos DW, Silva RR, Silva NF, Santos DA, et al Squamous cell carcinoma derived from chronic chromoblastomycosis in Brazil. Clin Infect Dis. 2015;60(10):1500–4. Epub 2015/02/15. doi: 10.1093/cid/civ104 . 25681378 10.1093/cid/civ104

[39] Rojas OC, Gonzalez GM, Moreno-Trevino M, Salas-Alanis J. Chromoblastomycosis by *Cladophialophora carrionii* associated with squamous cell carcinoma and review of published reports. Mycopathologia. 2015;179(1–2):153–7. Epub 2014/10/26. doi: 10.1007/s11046-014-9824-7 . 25344197 10.1007/s11046-014-9824-7

[40] Sharma N, Marfatia YS. Genital elephantiasis as a complication of chromoblastomycosis: A diagnosis overlooked. Indian J Sex Transm Dis. 2009;30(1):43–5. Epub 2009/01/01. doi: 10.4103/0253-7184.55486IJSTD-30-43 . 21938115 10.4103/2589-0557.55486 PMC3168040

[41] Azulay RD, Serruya J. Hematogenous dissemination in chromoblastomycosis: Report of a generalized case. Arch Dermatol. 1967;95(1):57–60. 6016308

[42] Carrion AL, Koppisch E. Observations on Dermatomycosis in Puerto Rico. Report on a Case of Chromoblastomycosis. Puerto Rico J Public Health Trop Med. 1933;9(2):169–90.

[43] Milam CP, Fenske NA. Chromoblastomycosis. Dermatol Clin. 1989;7(2):219–25. Epub 1989/04/01. . 2670367

[44] Gopal KVT, Ramani TV, Panda S, Laxmi PVBR. Disseminated chromoblastomycosis: Diffuse truncal involvement with hematogenous spread. Int J Health Allied Sci. 2012;1(3):194–6.

[45] Rajam RV, Kandhari KC, Thirumalachar MJ. Chromoblastomycosis caused by a rare yeast like dematiaceous fungus. Mycopathol Mycol Appl. 1958;9(1):5–19. Epub 1958/03/15. . 13541319 10.1007/BF02051408

[46] Muhammed K, Nandakumar G, Asokan KK, Vimi P. Lymphangitic chromoblastomycosis. Indian J Dermatol Venereol Leprol. 2006;72(6):443–5. Epub 2006/12/21. . 17179621 10.4103/0378-6323.29342

[47] Nair SP, Sarojini PA. Chromoblastomycosis resembling sporotrichosis. Ind J Dermatol Venereol Leprol. 1993;59:125–6.

[48] Verma GK, Verma S, Singh G, Shanker V, Tegta GR, Minhas S, et al A case of extensive chromoblastomycosis from North India. Braz J Microbiol. 2014;45(1):275–7. Epub 2014/06/21. doi: 10.1590/S1517-83822014005000025 . 24948945 10.1590/S1517-83822014005000025 PMC4059311

[49] Chowdhary A, Guarro J, Randhawa HS, Gene J, Cano J, Jain RK, et al A rare case of chromoblastomycosis in a renal transplant recipient caused by a non-sporulating species of Rhytidhysteron. Med Mycol. 2008;46(2):163–6. Epub 2008/03/08. doi: 10.1080/13693780701630420 . 18324495 10.1080/13693780701630420

[50] Dupont C, Duong TA, Mallet S, Mamzer-Bruneel MF, Thervet E, Bougnoux ME, et al Unusual presentation of chromoblastomycosis due to *Cladophialophora carrionii* in a renal and pancreas transplant recipient patient successfully treated with posaconazole and surgical excision. Transpl Infect Dis. 2010;12(2):180–3. Epub 2009/12/17. doi: 10.1111/j.1399-3062.2009.00477.x . 20002358 10.1111/j.1399-3062.2009.00477.x

[51] Franco A, Aranda I, Fernandez MJ, Arroyo MA, Navas F, Albero D, et al Chromomycosis in a European renal transplant recipient. Nephrol Dial Transplant. 1996;11(4):715–6. Epub 1996/04/01. . 8671869 10.1093/oxfordjournals.ndt.a027370

[52] Franco A, Aranda I, Navas F, Olivares J. Follow-up on a transplant recipient with chromomycosis. Nephrol Dial Transplant. 1997;12(4):852–3. Epub 1997/04/01. . 9141037 10.1093/ndt/12.4.852b

[53] Morales LA, Gonzalez ZA, Santiago-Delpin EA. Chromoblastomycosis in a renal transplant patient. Nephron. 1985;40(2):238–40. Epub 1985/01/01. . 3889680 10.1159/000183468

[54] Ogawa MM, Peternelli MP, Enokihara MM, Nishikaku AS, Goncalves SS, Tomimori J. Spectral Manifestation of Melanized Fungal Infections in Kidney Transplant Recipients: Report of Six Cases. Mycopathologia. 2016;181(5–6):379–85. Epub 2016/03/31. doi: 10.1007/s11046-016-0005-8 . 27025729 10.1007/s11046-016-0005-8

[55] Sooraj YS, Nainan GK, Eapen M, Immanuel AJ, Pillai RR. Chromoblastomycosis in a renal allograft recipient. Indian J Nephrol. 2013;23(3):235–6. Epub 2013/07/03. doi: 10.4103/0971-4065.111868 . 23814429 10.4103/0971-4065.111868 PMC3692156

[56] Verma P, Karmakar S, Pandhi D, Singal A, Yadav P, Khare S. Chromoblastomycosis Caused by *Cladophialophora bantiana* in a Renal Transplant Recipient From Delhi, India. Skinmed. 2015;13(3):251–4. Epub 2015/09/19. . 26380516

[57] Wackym PA, Gray GF Jr., Richie RE, Gregg CR. Cutaneous chromomycosis in renal transplant recipients. Successful management in two cases. Arch Intern Med. 1985;145(6):1036–7. Epub 1985/06/01. . 3890788

[58] Fuchs J, Pecher S. Partial suppression of cell mediated immunity in chromoblastomycosis. Mycopathologia. 1992;119(2):73–6. Epub 1992/08/01. . 1435950 10.1007/BF00443936

[59] Murthy R, Swain JP. Concurrent mycetoma and chromomycosis. Indian J Med Microbiol. 2011;29(4):437–9. Epub 2011/11/29. doi: 10.4103/0255-0857.90192 . 22120813 10.4103/0255-0857.90192

[60] Passeron T, Barberet P, Colbachini P, Hovette P, Lacour JP. [Concurrent mycetoma and chromomycosis: case report from Senegal]. Med Trop (Mars). 2003;63(6):614–6. Epub 2004/04/14. . 15077427

[61] Wortman PD. Concurrent chromoblastomycosis caused by *Fonsecaea pedrosoi* and actinomycetoma caused by *Nocardia brasiliensis*. J Am Acad Dermatol. 1995;32(2 Pt 2):390–2. Epub 1995/02/01. . 7829748 10.1016/0190-9622(95)90412-3

[62] Brygoo ER, Destombes P. Geographical distribution of chromoblastomycosis and its various pathogenic agents. Bull Soc Fr Mycol Med. 1975;4:181–3.

[63] Leslie DF, Beardmore GL. Chromoblastomycosis in Queensland: a retrospective study of 13 cases at the Royal Brisbane Hospital. Australas J Dermatol. 1979;20(1):23–30. Epub 1979/04/01. . 475694 10.1111/j.1440-0960.1979.tb00120.x

[64] Londero AT, Ramos CD. Chromomycosis: a clinical and mycologic study of thirty-five cases observed in the hinterland of Rio Grande do Sul, Brazil. The American journal of tropical medicine and hygiene. 1976;25(1):132–5. 1259077 10.4269/ajtmh.1976.25.132

[65] Simson FW. Chromoblastomycosis; some observations on the types of the disease in South Africa. Mycologia. 1946;38(4):432–49. Epub 1946/07/01. . 20992395

[66] Ameen M. Managing chromoblastomycosis. Trop Doct. 2010;40(2):65–7. Epub 2010/03/23. doi: 10.1258/td.2009.090264 . 20305094 10.1258/td.2009.090264

[67] Correia RT, Valente NY, Criado PR, Martins JE. Chromoblastomycosis: study of 27 cases and review of medical literature. An Bras Dermatol. 2010;85(4):448–54. Epub 2010/10/15. . 20944904 10.1590/s0365-05962010000400005

[68] Pradhan SV, Talwar OP, Ghosh A, Swami RM, Shiva Raj KC, Gupta S. Chromoblastomycosis in Nepal: a study of 13 cases. Indian J Dermatol Venereol Leprol. 2007;73(3):176–8. Epub 2007/06/15. . 17558050 10.4103/0378-6323.32741

