# Supplementary material for: Chromoblastomycosis in India: Review of 169 cases
Source: PLoS Negl Trop Dis. 2017 Aug 3;11(8):e0005534. doi: 10.1371/journal.pntd.0005534 (PMC5542425; doi:10.1371/journal.pntd.0005534)
Supplement: S1 Table — (DOCX) [file pntd.0005534.s001.docx]

| **S. No** | **Reference** | **Year** | **Age /Sex** | **Occupation** | **Location** | **Clinical presentation** | **Duration** | **History of trauma** | **Culture** | **Treatment** | **Outcome** |
| --- | --- | --- | --- | --- | --- | --- | --- | --- | --- | --- | --- |
| 1. | [1] | 1957 | 34/F | Agriculture | Assam | Nodular growth over vulva | 2 yrs | NA | *F.compactum (Hormonendrum compactum)* | NA | NA |
| 2. | [2] | 1957 | 35/M | Village preacher | Assam | Verrucous Lesions on right leg | 12 yrs | NA | *F.compactum (Hormonendrum compactum)* | NA | NA |
| 3. | [3] | 1958 | 7/M | NA | Punjab | Verrucous crusted on face, trunk and joints of extremities | 7yrs | NA | *E. spinifera* | Nystatin | Died |
| 4. | [4] | 1958 | NA | NA | Maharashtra | Papular lesion above umblicus | NA | NA | *Hormodendrum* sp. | NA | NA |
| 5. | [5] | 1966 | 50/M | Clerk | Karnataka | Cauliflower like growth on left forearm | 3 yrs | NA | Culture not done | NA | NA |
| 6. | [5] | 1966 | 30/M | Farmer | Karnataka | Scaly patch on left elbow | 14 yrs | yes | *F.compactum (Hormonendrum compactum)* | NA | NA |
| 7. | [6] | 1966 | 35/M | Labourer | Kerala | Warty plaques over lower half of left leg and sole of foot | 8 yrs | NA | *Fonsecae spp (Hormonendrum*  *spp)* | NA | NA |
| 8. | [7] | 1967 | 55/M | Toddy tapper | Telangana (earlier Andhra Pradesh) | Verrucous lesions on left leg, both thighs, left buttock, right hand +lympho nodes enlargement | 1 yr | no | *Cladosporium spp* | Potassium iodide | NA |
| 9. | [8] | 1967 | 30/M | NA | Uttrakhand | foot | NA | NA | *C. carrionii* | NA | NA |
| 10. | [8] | 1967 | 30/F | NA | Uttrakhand | Papillary and warty lesion on foot | NA | NA | *C.carrionii* | NA | NA |
| 11. | [9] | 1970 | 31/F | Housewife | Tamil Nadu | Papule on face, bridge of nose and left cheek | 5 yrs | No | *Cladosporium spp* | NA | NA |
| 12. | [9] | 1970 | 42/M | Cook | Tamil Nadu | plaque on dorsal aspect of right second toe | 4.5 yrs | yes | *F.compactum (Hormonendrum compactum)* | Surgical excision | Amputation  (cure) |
| 13. | [10] | 1972 | 25/M | Farmer | Andhra Pradesh | Hyperkeratotic, crusted lesions over the extremities, face and trunk+ inguinal lymph node enlarged | NA | NA | *F.pedrosoi*  *(Nonnodendrum pedrosoi)* | Iodides | Cure |
| 14. | [11] | 1973 | 43/M | Agriculturist | Kerala | Swelling over posterior aspect of right elbow joint | 2 yrs | no | Culture not done | NA | NA |
| 15. | [12] | 1974 | 33/M | Agriculturist | Karnataka | Hypertrophic  Verrucous plaques on left leg | 23 yrs | yes | *F.pedrosoi*  *(Phialophora*  *pedrosoi)* | Isonicotinoic acid hydrazide | Cure |
| 16. | [13] | 1975 | 40/M | Cultivator | West Bengal | Warty growth on dorsum of left foot and enlarged lymph nodes of groin | NA | yes | NA | Excision | Cure |
| 17. | [14] | 1977 | 40/M | Farmer | Haryana | Verrucous masses over right lower limb and both upper arms | 20 yrs | NA | Negative | Potassium iodide,isonex,  Thiabendazole+5% Salicyclic acid ointment | Initial cure but later patient defaulted |
| 18. | [15, 16] | 1978 and 1995 | 26/M  48 M | Agriculturist | Punjab | Plaque on face, both hands, dorsum of right foot and after 20 years involvement of gut + larynx + trachea | 28 yrs in total | No | *F.pedrosoi*  *(Hormodendrum pedrosoi)* | Potassium iodide + Amphotericin B +Thiabendazole +ketoconazole | Died |
| 19. | [15] | 1978 | 12/M | School boy | Punjab | plaques on face, right shoulder, right upper extremity, left thigh | 9 mnths | No | *C. carrionii* | Arnphotericin B | Not Cure |
| 20. | [17] | 1978 | 21/M | Compositor in press | Karnataka | swelling over Right parotid region | 20 days | NA | *F.pedrosoi*  *(Phialophora*  *pedrosoi)* | Potassium iodide+  Griseofulvin+superficial x-ray therapy | Cure |
| 21. | [18] | 1981 | 40/M | Coal filler | Andhra Pradesh | Verrucous masses over lower 2/3 leg +enlarged lymph nodes | 12 yrs | yes | *F.pedrosoi*  *(Phialophora*  *pedrosoi)* | Hamycin and antihistamines | Cure |
| 22. | [18] | 1981 | 43/M | NA | Andhra Pradesh | Verrucous lesion over right foot | 2 yrs | NA | Contaminants grown | Hamycin and antihistamines | Cure |
| 23. | [18] | 1981 | 60/M | NA | Telangana (earlier Andhra Pradesh) | Warty lesion on left foot | 6 Mnths | Yes | Contaminants grown | Hamycin and antihistamines + excision | Cure |
| 24. | [19] | 1984 | 10F | NA | Pondicherry | plaque on face,trunk and exremities | 1 Yr | NA | *E. jeanselmei* | griseofulvin +5 flurouracil + thiabendazole + amphotericin B | Lost to follow up |
| 25. | [20] | 1985 | 35/M | NA | Chandigarh | Erythematous and verrucous lesions from dorsum of foot to lower half of right thigh + enlarged inguinal lymph nodes | 20 yrs | no | *C. carrionii* | NA | NA |
| 26. | [21] | 1986 | 68/M | Carpenter | Meghalya | verrucous plaques on the leg and penis | NA | NA | *F. pedrosoi* | mebendazole | Not cured |
| 27. | [22] | 1988 | 50/M | NA | Kerala | Verrucous lesion on left knee and left upper arm | 6 mnths | no | *C. carrionii* | ketoconazole | Cure |
| 28. | [23] | 1988 | 47/M | NA | Tamil Nadu | verrucous lesions on the right upper limb and metastasis to axillary lymph node | 33 yrs | NA | *C. cladosporioides* | amphotericin B and 5fluorouracil | Cure  Died due to carcinoma |
| 29. | [24] | 1989 | 50/M | Farmer | Himachal Pradesh | nodular lesions on the foot | 6 yrs | No | *P. verrucosa* | Potassium iodide | Cure |
| 30. | [25] | 1991 | 42/F | NA | Kerala | Verrucous nodules and plaques on the face, neck, trunk and limbs and enlarged lymph nodes | 1 yr | No | *C. carrionii* | intravenous amphotericin and oral ketoconazole | Partial cure |
| 31. | [26] | 1991 | 12/M | School boy | Chandigarh | Plaque over left side of face and right leg | 4 yrs | NA | *F. pedrosoi* | Ketoconazole,  itraconazole | Lost to follow up |
| 32. | [26] | 1991 | 43/M | NA | Chandigarh | Nodular and plaque lesions on both lower limbs and both thighs | 22yrs | NA | *F. pedrosoi* | Amphotericn ketoconazole,  Potassium iodide  itraconazole | Cure |
| 33. | [27] | 1992 | 21/M | NA | Kerala | Plaque on lumbar region | NA | NA | *C.carrioni* | surgical excision | Cure |
| 34. | [28] | 1993 | 21/M | NA | Punjab | nodular lesions on right hand and forearm | 1 Yr | No | Negative | Potassium iodide | Cure |
| 35. | [29] | 1993 | 20/F | NA | Maharashtra | Warty growth on right leg | 8 yrs | NA | *F. compactum* | NA | NA |
| 36. | [30] | 1994 | 40/M | agriculturist | Tamil Nadu | nodular pigmented verrucous lesions over both legs and  feet | 5 yrs | NA | *P.verrucosa* | NA | NA |
| 37. | [31] | 1995 | NA | NA | Karnataka | Lesion on leg | NA | NA | NA | Cryotherapy | cure |
| 38. | [31] | 1995 | NA | NA | Karnataka | Plaque on lower leg | NA | NA | NA | Cryotherapy,  CO_2_ laser | cure |
| 39. | [32] | 1997 | 45/M | NA | Jammu and Kashmir | erythematous  scaly plaque on both buttocks | 33 yrs | Not recalled | *C. carrionii.* | Ketoconazole | NA |
| 40. | [32] | 1997 | 40/M | Farmer | Bihar | a small  lesion on left elbow | 5 yrs | NA | *Fonsecaea spp* | Surgical excision | Cure |
| 41. | [32] | 1997 | 30/F | NA | Uttar Pradesh | verrucous lesion on the right foot | 6 yrs | No | *F. pedrosoi* | Nil | Lost to follow up |
| 42. | [32] | 1997 | 40/M | NA | Assam | erythematous hypertrophic plaque on  right leg and ankle | 13 yrs | yes | *F. pedrosoi* | amphotericin B and 5-fluorocytosine | Cure |
| 43. | [33] | 1998 | 42/F | NA | Kerala | papules on the right earlobe, right check and mucosa of the nostrils | 3 mnths | yes | *C. carrionii* | Ketoconazole,  Itraconazole | Lost to follow up |
| 44. | [34] | 1999 | 8/M | NA | Maharashtra | Plaques on both sole and left palm | 4 yrs | NA | *F. pedrosoi* | NA | NA |
| 45. | [35] | 1999 | 80/M | Farmer | Himachal Pradesh | verrucous plaque over the dorsum of the left hand, wrist, forearm + pleural effusion and enlarged lymph nodes | 12 yrs | NA | *F.pedrosoi* | potassium iodide + Fluconazole | Cure |
| 46. | [35] | 1999 | 47/M | Farmer | Himachal Pradesh | verrucous crusted lesions over the toes, dorsum of the foot | 25 yrs | NA | *F. pedrosoi* | potassium iodide+ Cryotherapy+ fluconazole+itraconazole | Cure |
| 47. | [35] | 1999 | 48/M | Farmer | Himachal Pradesh | Crusted verrucous lesions on fingers, hand, wrist, and forearm and enlarged lymph nodes | 7 yrs | Yes | *C. carrioni* | potassium iodide+ fluconazole+itraconazole | Cure |
| 48. | [35] | 1999 | 27/M | Horticulturist | Himachal Pradesh | erythematous plaque on left index finger | 10 Months | yes | *P. verrucosum* | Surgical excision+  Itraconazole | Cure |
| 49. | [36] | 2000 | 38/M | Farmer | Maharashtra | nodular swelling over the left palm | 4 yrs | yes | *Phialophora spp* | Itraconazole,  Cryotherapy | Cure |
| 50. | [37] | 2002 | 62/M | NA | Delhi | Cauliflower  like growth over left foot and ankle | 12 yrs | yes | Culture not done | Itraconazole | Cure |
| 51. | [38] | 2005 | 70/M | Agriculturist | Bangalore | Verrucous lesion on medial aspect of left leg | 1 yr | yes | *Cladophialophora spp* | itraconazole +Surgical excision | Cure |
| 52. | [39] | 2006 | 40/M | Farmer | Kerala | Multiple nodules on right leg and foot | 1 yr | yes | *F. pedrosoi* | itraconazole | Cure  died due to ishemic heart disease |
| 53. | [40] | 2006 | 65/F | NA | Orissa | Verrucous growth on right leg | 1 yr | NA | Culture not done | NA | NA |
| 54. | [41] | 2007 | 45/M | Farmer | Assam | warty growths  on the right leg, knee and thigh | 35 yrs | yes | *F. pedrosoi* | NA | NA |
| 55. | [42] | 2007 | 42/M | Farmer | Himachal Pradesh | plaque on right foot | 5 yrs | yes | *F. pedrosoi* | itraconazole + potassium iodide | cure |
| 56. | [43] | 2008 | 54/M | NA | Maharashtra | warty growth on the medial aspect of left thigh | 1 yr | no | *F. pedrosoi* | Itraconazole | Cure |
| 57. | [44] | 2008 | 50/M | NA | Delhi | noduloulcerative lesions on his left leg +lymphadenopathy | 2 mnths | Cannot recalled | *Rhytidhysteron rufulum* | Itraconazole | Partial cure |
| 58. | [42] | 2009 | 20/M | Truck driver | Gujarat | plaques over lower abdomen, face, neck,thighs and axilla + genital elephantiasis | 6mnths | no | Culture not done | Fluconazole + Cryotherapy | Partial cure |
| 59. | [45] | 2009 | 60/M | Farmer | Karnataka | verrucous lesions over the right leg | 5 yrs | Cannot recalled | *C. carrionii* | NA | NA |
| 60. | [46] | 2010 | 81/M | Priest | Karnataka | verrucous lesions over the right leg | 5 yrs | No | *C. carrionii.* | Itraconazole | Cure |
| 61. | [47] | 2010 | 16/M | Worker in a tea garden | Assam | papulated plaques on both upper and lower extremities, trunk, both the ears, scalp and genitalia | 6mnths | yes | *C. carrionii*. | Fluconazole | Cure |
| 62. | [47] | 2010 | 55/M | Worker in a tea garden | Assam | cauliflower like mass in his left leg | 9 yrs | yes | *F. pedrosoi* | Fluconazole | Cure |
| 63. | [48] | 2010 | 46/M | NA | West Bengal | Hyperpigmented, verrucous over chin and malar region | 7 mnths | No | Culture not done | Itraconazole | Lost to follow up |
| 64. | [49] | 2011 | 29/M | NA | Orissa | ulcerated warty growth over chin | 1 yr | No | *F. pedrosoi* | Itraconazole | Cure |
| 65. | [50] | 2011 | 9/M | NA | Andhra Pradesh | necrotic, hyperkeratotic verrucous plaque on left arm | 18 mnths | Yes | *C. carrionii*. | itraconazole + terbinafine | Cure |
| 66. | [51] | 2011 | 24/M | NA | Maharashtra | verrucous papules on right arm in leprosy pt | 5 mnths | Yes | *C. carionii* | Itraconazole | Cure |
| 67. | [52] | 2011 | 38/M | Farmer | Chhattisgarh | verrucose and discharging  nodules on left leg + mycetoma | 9 yrs | NA | *F. pedrosoi* | Itraconazole | lost to follow up. |
| 68. | [53] | 2011 | 37/M | Labourer | Delhi | nodule over lateral malleolus of left leg | 3 yrs | no | *C. carionii* | Surgical excision +itraconazole | Cure |
| 69. | [54] | 2012 | 67/F | Farmer | Himachal Pradesh | Erythematous plaque on right upper lip and lower third of right cheek | 7 mnths | no | *F. pedrosoi* | Itraconazole +potassium iodide+Terbenafine + Cryotherapy | Cure |
| 70. | [54] | 2012 | 57/F | NA | Himachal Pradesh | Ulcer on cheek and nose | 1yr | No | *F. pedrosoi* | potassium iodide ,Itraconazole +Terbenafine + Cryotherapy | Cure |
| 71. | [55] | 2012 | 35/F | Farmer | Karnataka | Phagedenic ulcer on the face | 20 yrs | yes | *F. pedrosoi* | fluconazole | Cure |
| 72. | [56] | 2012 | 50/M | agricultural worker | West Bengal | hyperpigmented  verrucous plaques in right arm | 1 and half years | Could not recalled | *F. pedrosoi* | Itraconazole | Cure |
| 73. | [57] | 2012 | 27/M | Bus driver | Andhra Pradesh | plaque over the lower back, posterior aspect of right  lower thigh, left shin, the right cheek, forehead, chin, and  extensor aspect of both elbows+ lymphadenopathy | 6 mnths | no | *C. carrionii*. | Itraconazole+  Terbenafine | Cure |
| 74. | [58] | 2012 | 58/M | NA | Maharashtra | Plaque lesion on head and neck | 1 mnth | no | *F. compacta* | NA | NA |
| 75. | [59]  35 Cases | 2012 | 25-69/  (30) males  (5)  females | agricultural tasks (21) | Kerala | verrucous plaques (31)  hyperkeratotic  plaques with scaling (3)  nodular cauliflower like growth (1) | NA | 14 recalled | *F. pedrosoi* (24).  *F. dermatitidis* (3)  *F. compactum* (2)  *P. verrucosa* (1)  *Cladosporium* spp (1). | potassium iodide,  Itraconazole,  Terbenafine | NA |
| 76. | [60] | 2013 | 50/F | NA | Kerala | ulcero‑proliferative lesion over the right heel | 1 month | NA | NA | Itraconazole | Cure |
| 77. | [61] | 2013 | 45/M | Farmer | Assam | warty  growths over the left foot | 1 yr | yes | *F. pedrosoi* | itraconazole +  terbinafine | Cure |
| 78. | [62] | 2013 | 27/F | NA | Maharashtra | erythematous  plaque over right maxillary area | 1 yr | no | *F. pedrosoi* | itraconazole +  terbinafine | Cure |
| 79. | [62] | 2013 | 55/M | NA | Maharashtra | itchy nodular lesion over lateral aspect of right lower lip | 8 mnths | no | *F. pedrosoi.* | itraconazole +  terbinafine | Cure |
| 80. | [63] | 2013 | 55/F | tea garden worker | West Bengal | verrucous  plaque on right arm | NA | NA | NA | NA | NA |
| 81. | [64] | 2013 | 54/M | Agriculturist | West Bengal | Verrucous lesions on eft leg | 10 yrs | No | *F. pedrosoi* | itraconazole | Cure |
| 82. | [64] | 2013 | 46/M | Gardener | West Bengal | itchy scaly plaque  on the right forearm | 3 yrs | NA | *F. pedrosoi* | itraconazole | Cure |
| 83. | [65] | 2013 | 74/M | Farmer | Maharashtra | Ulcer on medial aspect of right foot | 2 yrs | yes | Culture not done | NA | Cure |
| 84. | [66] | 2014 | 51/M | Farmer | Himachal Pradesh | veruccous to nodular lesions on right leg and thigh + lymphadenopathy | 5 yrs | no | *F. pedrosoi.* | Potassium iodide itraconazole terbenfine Cryotherapy | Cure |
| 85. | [67] | 2014 | 26/F | Housewife | Uttar Pradesh | Calcified mass in left nasal cavity | 2 yrs | no | Negative | itraconazole | Cure |
| 86. | [68] | 2014 | 32/M | Farmer | Rajasthan | verrucous plaques on right forearm | 3 yrs | NA | *C.carrionii* | itraconazole | Cure |
| 87. | [69] | 2014 | 35/M | Farmer | Tamil Nadu | Verrucous nodules and cauliflower growth on right foot | 10-15 yrs | NA | *F. pedrosoi* | Itraconazole +Terbenafine+surgical excision followed by amputation | Not cured |
| 88. | [70] | 2015 | 55/M | Farmer | Assam | verrucous plaques and  nodules on anterolateral left leg and knee | 32 yrs | Could not recalled | *C. cladosporioides* | itraconazole and surgical debridement | Cure |
| 89. | [70] | 2015 | 58/M | Farmer | Assam | plaque over the dorsum of right  foot | 1 Yr | no | *B. cynodontis* | itraconazole | Lost to follow up |
| 90. | [71] | 2015 | 38/F | Housewife | Delhi | Warty lesion on left lower leg | 20 yrs | Could not recalled | *C.carrionii* | itraconazole | Cure |
| 91. | [72] | 2015 | 44/M | Livestock supervisor | Madhya Pradesh | Verrucous plaque over the medial  aspect of right thigh in leprosy tt pt | 2 yrs | No | Culture not done | itraconazole | Cure |
| 92. | [73] | 2015 | 36/M | Industrial worker | Madhya Pradesh | papules on the lateral aspect of lower part of his left forelimb | 6 mnths | no | *F. pedrosoi* | Itraconazole+  terbenafine | Cure |
| 93. | [74] | 2015 | 40/F | NA | Telangana | Nodular lesion on both thighs and legs | 1mnth | NA | NA | NA | NA |
| 94. | [75] | 2015 | 63/F | Housewife | Telangana | verrucous plaques over the dorsum of the tongue | NA | no | NA | Itraconazole | Cure |
| 95. | [76] | 2015 | 40/M | NA | Maharashtra | Ulcer over right knee | NA | yes | *C. carrionii* | Itraconazole | Cure |
| 96. | [77] | 2015 | 57/M | Agriculturist | Kerala | Hypertrophic plaque on the right lower limb | 2 mnths | No | *F. pedrosoi* | Itraconazole+  cryotherapy | Cure |
| 97. | [77] | 2015 | 58/M | NA | Kochi | scaly lesion on right foot | 6 mnths | NA | *F. pedrosoi* | Itraconazole+  cryotherapy | Cure |
| 98. | [78] | 2015 | 45/M | Accountant | Delhi | plaque on the little finger of his left hand | 3 mnths | no | *C. bantiana* | Terbinafine+ excision | Lost to follow up |
| 99. | [79] | 2015 | 65/M | NA | Delhi | plaques over the right knee | 6 mnths | NA | NA | Itraconazole | NA |
| 100. | [80] | 2015 | NA | Agriculture | West Bengal | Plaque on left forearm | NA | NA | *F. pedrosoi* | traconazole | Cure |
| 101. | [80] | 2015 | NA | Agriculture | West Bengal | Plaque on medial aspect of left ankle | NA | NA | Negative | SSKI + debridement + Itraconazole | Cure |
| 102. | [80] | 2015 | NA | Agriculture | West Bengal | Plaque on right lower thigh, knee | NA | NA | *F. pedrosoi* | oral Itraconazole +SSKI | Partial cure |
| 103. | [80] | 2015 | NA | Agriculture | West Bengal | Plaque on front of right lower thigh | NA | NA | *Cladosporium spp.* | Itraconazole+ debridement | Cure |
| 104. | [80] | 2015 | NA | Agriculture | West Bengal | Plaque on right upper abdomen | NA | NA | Negative | Itraconazole | Partial cure |
| 105. | [81] | 2015 | 20-50 yrs/M N=6 | Farmer (3)  Wood cutter(1)  Manual labourer(1)  Unemployed(1) | Assam(5)  Arunachal  Pradesh (1) | Verrucous lesions on  Lower limb(5)  Upper limb(1) | 1-5 yrs | Yes(1)  No (5) | *C. cladosporioides* (*n* = 3),  *C. lunata* (*n* = 1)  *B. spicifera* (*n* = 1) | NA | NA |
| 106. | [82] | 2016 | 45/M | agriculturist | Kerala | Verrucous plaques nodules on left lower limb | 4 yrs | yes | *F. pedrosoi* | itraconazole | Cure |
| 107. | [83] | 2016 | 58/M | Rubber tapper | Karnataka | Lesion on left index finger | 2 yrs | no | *Fonsecaea spp* | fluconazole | Cure |
| 108. | [83] | 2016 | 40/F | Manual labourer | Karnataka | Raised lesion on left leg | 2 yrs | no | *Fonsecaea spp* | Fluconazole | Cure |
| 109. | [83] | 2016 | 46/M | Rubber tapper | Karnataka | Raised lesion on left little finger | 9 mnths | no | *Fonsecaea spp* | Terbinafine+  cryotherapy | Cure |
| 110. | [83] | 2016 | 44/M | Rubber tapper | Karnataka | Left middle finger | 1 yr | no | *Fonsecaea spp* | Fluconazole | Cure |
| 111. | [83] | 2016 | 35/F | Housewife | Karnataka | Raised lesion on right foot | 18 mnths | no | *Fonsecaea spp* | Terbinafine | Cure |
| 112. | [83] | 2016 | 58/M | Manual labourer | Karnataka | Raised lesion on left leg | 5 yrs | no | *Fonsecaea spp* | Itraconazole,  Fluconazole | Cure |
| 113. | [83] | 2016 | 50/M | Manual labourer | Karnataka | Raised lesion on left foot | 20 yrs | no | NA | Itraconazole,  Fluconazole,  Terbinafine,  Potaasium iodide | Cure |
| 114. | [83] | 2016 | 38/M | Autodriver | Karnataka | Verrucous plaque on left heel | 2 yrs | no | NA | Fluconazole+  cryotherapy | Cure |
| 115. | [83] | 2016 | 67/M | Manual labourer | Karnataka | Right foot dorsum | 4 yrs | no | NA | Fluconazole | Cure |
| 116. | [83] | 2016 | 74/M | NA | Karnataka | Reddish lesion on left knee | 5 yrs | no | NA | Itraconazole,  Terbinafine | Cure |
| 117. | [83] | 2016 | 64/F | Rubber tapper | Karnataka | Left medial malleolus | 3 yrs | no | NA | Terbinafine | Cure |
| 118. | [83] | 2016 | 40/F | Housewife | Karnataka | Foot | 6 mnths | no | NA | Terbinafine  + potassium iodide | Cure |
| 119. | [83] | 2016 | 50/M | Manual labourer | Karnataka | knee | 2 mnths | no | NA | Terbinafine | Cure |
| 120. | [83] | 2016 | 38/F | agriculture | Karnataka | ankle | 5 mnths | no | NA | Terbinafine | Cure |
| 121. | [83] | 2016 | 64/M | agriculture | Karnataka | Left leg | 1 yr | no | NA | Terbinafine | Cure |
| 122. | [83] | 2016 | 70/M | Rubber tapper | Karnataka | foot | 2 yrs | no | NA | Terbinafine | Cure |
| 123. | [84] | 2016 | 55/M | Tea gardener | West Bengal | Hypopigmented,  ulcerated cutaneous lesion over mid-shin part of left lower limb | 4 mnths | no | *F. pedrosoi* | Terbinafine+  Surgical excision | Cure |
| 124. | [85]  N=3 | 2016 | NA | NA | Himachal Pradesh(3) | NA | NA | NA | NA | NA | NA |
| 125. | [86] | 2016 | 54/M | Farmer | Karnataka | erythematous plaque over right axilla | 1 yr | NA | *Fonsecaea spp* | itraconazole +  cryotherapy | cure |
| 126. | [86] | 2016 | 37/M | Cook | Karnataka | plaque over axilla | 2 yrs | no | Negative | Itraconazole | cure |
| 127. | Present case | 2016 | 27/F | Housewife | Uttarakhand | plaques on the anterior aspect of lower 1/3 of right leg. | 8 Yrs | no | *F. pedrosoi* | Terbinafine, itraconazole,  Ketoconazole,  Amphotericin B +cryosurgery | cure |
| 128. | Present case | 2016 | 70/M | Farmer | Uttarakhand | ulceroproliferative lesion on the dorsum of the right foot | 4 yrs | yes | *Cladosporium tenuissimum* | itraconazole | cure |

NA: not available

**REFERENCES:**

1. Kakoti LM, Dey NC. Chromoblastomycosis in India. J Indian Med Assoc. 1957;28(8):351-5. Epub 1957/04/16. PubMed PMID: 13429115.

2. Thomas E, Job CK, Hadley GG. Chromoblastomycosis. Indian J Med Sci. 1957;11(8):570-3. Epub 1957/08/01. PubMed PMID: 13474771.

3. Rajam RV, Kandhari KC, Thirumalachar MJ. Chromoblastomycosis caused by a rare yeast like dematiaceous fungus. Mycopathol Mycol Appl. 1958;9(1):5-19. Epub 1958/03/15. PubMed PMID: 13541319.

4. Gokhale BB, Thirumalachar MJ, Padhye AS. A case of black papulosquamous skin infection by *Hormodendron* species.(Abstract). Symposium of fungal diseases in India. Calcutta Bull Cal School Trop Med. 1959;7:41.

5. Dube B, Dube R. Chromoblastomycosis in India. A review and report of two new cases. Indian J Med Sci. 1966;20(1):10-6. Epub 1966/01/01. PubMed PMID: 5904791.

6. Meenakshi LV, Balakrishnan P, Kannankutty M, Mathew KT, Ananthanarayan R. Chromoblastomycosis. A short review with report of a case. Indian Pract. 1966;19(7):449-52. Epub 1966/07/01. PubMed PMID: 5915645.

7. Murty KR, Vasu BH. Clinical features of an unusual case of chromoblastomycosis. Indian J Dermatol. 1967;12(2):55-6. Epub 1967/01/01. PubMed PMID: 6037977.

8. Mohapatra LN, Sood VK, Grueber HLE. Chromoblastomycosis in India and Nepal. Mycoses. 1967;10(8):309-14.

9. Bhaktaviziam A, Mathai R, Mammen A, Bhaktaviziam CA. Chromoblastomycosis in India. Indian J Dermatol Venerol. 1970;36:185-8.

10. Sivaramakrishna PN, Baburaja KK. Chromoblastomycosis. Ind J Dermatol Venereol. 1972;38:78-82.

11. Venkataramaiah NR, Rao RV, Hafiz FA, Bhat S, Venugopal N. Chromoblastomycosis. Ind J Dermatol Venereol. 1973;39(5):207-8.

12. Nagarkatti PS, Ramachandraiah V, Rajashekaraiah HK. Chromoblastomycosis (A case report). Ind J Dermatol Venereol. 1974;40(6):265-7.

13. Mukherjee B, Sengupta D, Bhattacharyya KK. Chromoblastomycosis. J Indian Med Assoc. 1975;64(12):338-40. Epub 1975/06/16. PubMed PMID: 1184986.

14. Verma KC, Chaudhary SD, Chugh TD, Bhargava N. Chromoblastomycosis (A case report). Ind J Dermatol Venereol. 1977;43(1):35-7.

15. Kumar K, Sarin RC. Chromoblastomycosis Ind J Dermatol Venereol. 1978;44(5):310-5.

16. Bharti R, Malhotra SK, Bal MS, Sharma K. Chromoblastomycosis. Indian J Dermatol Venereol Leprol. 1995;61(1):54-5. Epub 1995/01/01. doi: ijdvl_1995_61_1_54_4133 [pii]. PubMed PMID: 20952879.

17. Shivananda PG, Ganesan MV, Pai KR, Rao KNA. Chromomycosis in man in an unusual site. Indian J Dermatol Venereol Leprol. 1978;44(6):369-71.

18. Radhakrishnamurthy K. Chromomycosis due to *Phialophora pedrosoi*. Indian J Dermatol Venereol Leprol. 1981;47(5):281-4

19. Lal S, Garg BR, Rao RS, Sharma S, Veliath AJ. Chromomycosis caused by *Exophiala jeanselmei*. Ind J Dermatol Venereol. 1984;50:119-21.

20. Sharma KV, Kumar B, Kaur I, Kaur S. Side lab diagnosis of chromoblastomycosis. Ind J Dermatol Venereol Leprol. 1985;51(3):157-9.

21. Battu V, Ramam M, Pasricha JS, Mohapatra LN. Chromomycosis with some unusual features. Ind J Dermatol Venereol Leprol. 1986;52(4):181-3.

22. Pavithran K. Chromoblastomycosis in a residual patch of leprosy. Indian J Lepr. 1988;60(3):444-7. Epub 1988/07/01. PubMed PMID: 3198964.

23. Jacob M, Mathai R, Prasad PVS, Bhaktaviziam A. Chromoblastomycosis with squamous cell carcinoma. Ind J Dermatol Venereol Leprol. 1988;54(6):314-7.

24. Sharma NL, Sharma RC, Gupta ML, Singh P, Arora VK. Cutaneous chromomycosis with pulmonary geotrichosis. Ind J Dermatol Venereol Leprol. 1989;55(5):331-3.

25. Pavithran K. Disseminated chromoblastomycosis. Ind J Dermatol Venereol Leprol. 1991;57(3):155-6.

26. Kumar B, Kaur I, Chakrabarti A, Sharma VK. Treatment of deep mycoses with itraconazole. Mycopathologia. 1991;115(3):169-74. Epub 1991/09/01. PubMed PMID: 1660959.

27. Pavithran K. Chromoblastomycosis masquerading as tuberculoid leprosy. Int J Lepr Other Mycobact Dis. 1992;60(4):657-8. Epub 1992/12/01. PubMed PMID: 1299721.

28. Nair SP, Sarojini PA. Chromoblastomycosis resembling sporotrichosis. Ind J Dermatol Venereol Leprol. 1993;59:125-6.

29. Deshpande S, Sahni S, Murti P. Case reports of chromomycosis. Indian J Pathol Microbiol. 1993;36(4):469-73. Epub 1993/10/01. PubMed PMID: 8157318.

30. Harshan V. Chromoblastomycosis due to *Phialophora verrucosa*. Ind J Dermatol Venereol Leprol. 1994;60(2):95-6.

31. Shenoi SD, Srinivas CR. Chromoblastomycosis. Indian J Dermatol Venereol Leprol. 1995;61(4):245-6. Epub 1995/07/01. doi: ijdvl_1995_61_4_245_4232 [pii]. PubMed PMID: 20952979.

32. Rajendran C, Ramesh V, Misra RS, Kandhari S, Upreti HB, Datta KK. Chromoblastomycosis in India. Int J Dermatol. 1997;36(1):29-33. Epub 1997/01/01. PubMed PMID: 9071611.

33. Pavithran K. Chromoblastomycosis simulating lepromatous leprosy. Int J Lepr Other Mycobact Dis. 1998;66(1):59-61. Epub 1998/06/06. PubMed PMID: 9614844.

34. Lakshmi TSS, Rao G, Vijay AS. Chromoblastomycosis masquerading as palmo-plantar psoriasis. Ind J Dermatol Venereol Leprol. 1999;65(2):83-4.

35. Sharma NL, Sharma RC, Grover PS, Gupta ML, Sharma AK, Mahajan VK. Chromoblastomycosis in India. Int J Dermatol. 1999;38(11):846-51. Epub 1999/12/03. doi: ijd820 [pii]. PubMed PMID: 10583618.

36. Bhise PR, Sony PR. Chromomycosis: Subcutaneous cystic type. Ind J Dermatol Venereol Leprol. 2000;66(2):99-100.

37. Sayal SK, Prasad GK, Jawed KZ, Sanghi S, Satyanarayana S. Chromoblastomycosis. Indian J Dermatol Venereol Leprol. 2002;68(4):233-4. Epub 2007/07/28. PubMed PMID: 17656949.

38. Vijaya D, Kumar BH. Chromoblastomycosis. Mycoses. 2005;48(1):82-4. Epub 2005/02/01. doi: MYC1060 [pii]

10.1111/j.1439-0507.2004.01060.x. PubMed PMID: 15679674.

39. Muhammed K, Nandakumar G, Asokan KK, Vimi P. Lymphangitic chromoblastomycosis. Indian J Dermatol Venereol Leprol. 2006;72(6):443-5. Epub 2006/12/21. PubMed PMID: 17179621.

40. Mohanty L, Mohanty P, Padhi T, Samantray S. Verrucous growth on leg. Indian J Dermatol Venereol Leprol. 2006;72(5):399-400. Epub 2006/10/20. PubMed PMID: 17050950.

41. Rasul ES, Hazarika NK, Sharma A, Borua PC, Sen SS. Chromoblastomycosis. J Assoc Physicians India. 2007;55:149-51. Epub 2007/06/19. PubMed PMID: 17571747.

42. Sharma NL, Sharma VC, Mahajan V, Shanker V, Sarin S. Chromoblastomycosis with underlying osteolytic lesion. Mycoses. 2007;50(6):517-9. Epub 2007/10/20. doi: MYC1398 [pii]

10.1111/j.1439-0507.2007.01398.x. PubMed PMID: 17944717.

43. Momin YA, Raghuvanshi SR, Lanjewar DN. Cutaneous chromoblastomycosis. Bombay Hosp J. 2008;50:299-301.

44. Chowdhary A, Guarro J, Randhawa HS, Gene J, Cano J, Jain RK, et al. A rare case of chromoblastomycosis in a renal transplant recipient caused by a non-sporulating species of Rhytidhysteron. Med Mycol. 2008;46(2):163-6. Epub 2008/03/08. doi: 791067645 [pii]

10.1080/13693780701630420. PubMed PMID: 18324495.

45. Bhagwat P, Tophakhane R, Kudligi C, Noronha T. Multiple asymptomatic verrucous plaques over the legs. Ind J Dermatol Venereol Leprol. 2009;76(1):86.

46. Namratha N, Nadgir S, Kale M, Rathod R. Chromoblastomycosis due to *Cladosporium carrionii*. J Lab Physicians. 2010;2(1):47-8. Epub 2010/01/01. doi: 10.4103/0974-2727.66704

JLP-2-47 [pii]. PubMed PMID: 21814409.

47. Sharma A, Hazarika NK, Gupta D. Chromoblastomycosis in sub-tropical regions of India. Mycopathologia. 2010;169(5):381-6. Epub 2010/01/23. doi: 10.1007/s11046-009-9270-0. PubMed PMID: 20094803.

48. De A, Gharami RC, Datta PK. Verrucous plaque on the face: what is your diagnosis? Dermatol Online J. 2010;16(1):6. Epub 2010/02/09. PubMed PMID: 20137748.

49. Mishra A, Tripathi K, Biswal P, Rath J. Chromoblastomycosis of chin masquerading as facial wart. Indian J Pathol Microbiol. 2011;54(1):221-2. Epub 2011/03/12. doi: IndianJPatholMicrobiol_2011_54_1_221_77422 [pii]

10.4103/0377-4929.77422. PubMed PMID: 21393930.

50. Pradeepkumar NS, Joseph NM. Chromoblastomycosis caused by *Cladophialophora carrionii* in a child from India. J Infect Dev Ctries. 2011;5(7):556-60. Epub 2011/07/29. PubMed PMID: 21795827.

51. Apte G, Gedam JR, Poojary S, Nagpur NG, Pai VV, Ganapathi R. Chromoblastomycosis in a case of borderline lepromatous leprosy with recurrent type II lepra reaction. Lepr Rev. 2011;82(3):310-5. Epub 2011/12/01. PubMed PMID: 22125940.

52. Murthy R, Swain JP. Concurrent mycetoma and chromomycosis. Indian J Med Microbiol. 2011;29(4):437-9. Epub 2011/11/29. doi: IndianJMedMicrobiol_2011_29_4_437_90192 [pii]

10.4103/0255-0857.90192. PubMed PMID: 22120813.

53. Azad K, Khanna G, Capoor MR, Gupta S. *Cladophialophora carrionii*: an aetiological agent of cutaneous chromoblastomycosis from a non-endemic area, North India. Mycoses. 2011;54(4):e217-9. Epub 2009/12/24. doi: MYC1817 [pii]

10.1111/j.1439-0507.2009.01817.x. PubMed PMID: 20028462.

54. Verma S, Verma GK, Singh G, Kanga A, Sharma V, Gautam N. Facial chromoblastomycosis in sub-Himalayan region misdiagnosed as cutaneous leishmaniasis: brief report and review of Indian literature. Dermatol Online J. 2012;18(10):3. Epub 2012/11/06. PubMed PMID: 23122010.

55. Naveen KN, Shetty PC, Naik AS, Pai VV, Hanumanthayya K, Udupishastry D. Chromoblastomycosis presenting as a phagedenic ulcer on the face. Int J Dermatol. 2012;51(5):576-8. Epub 2011/09/20. doi: 10.1111/j.1365-4632.2011.05088.x. PubMed PMID: 21923692.

56. Bandyopadhyay A, Majumdar K, Gangopadhyay M, Banerjee S. Cutaneous Chromoblastomycosis Mimicking Tuberculosis Verrucosa Cutis: Look for Copper Pennies! Turkish J Pathol. 2012;31(3):223-5.

57. Gopal KVT, Ramani TV, Panda S, Laxmi PVBR. Disseminated chromoblastomycosis: Diffuse truncal involvement with hematogenous spread. Int J Health Allied Sci. 2012;1(3):194-6.

58. Angadi KM, Misra RN, Gandham NR, Moumita S, Vyawahare CR, Singhania SS, et al. Chromoblastomycosis: a rare case of infection by *Fonsecaea compacta* from Western Maharashtra, India. International J Microbiol Res. 2012;4(9):330-1.

59. Chandran V, Sadanandan SM, Sobhanakumari K. Chromoblastomycosis in Kerala, India. Indian J Dermatol Venereol Leprol. 2012;78(6):728-33. Epub 2012/10/19. doi: ijdvl_2012_78_6_728_102366 [pii]

10.4103/0378-6323.102366. PubMed PMID: 23075642.

60. Sooraj YS, Nainan GK, Eapen M, Immanuel AJ, Pillai RR. Chromoblastomycosis in a renal allograft recipient. Indian J Nephrol. 2013;23(3):235-6. Epub 2013/07/03. doi: 10.4103/0971-4065.111868

IJN-23-235 [pii]. PubMed PMID: 23814429.

61. Roy AD, Das D, Deka M. Chromoblastomycosis - A clinical mimic of squamous carcinoma. Australas Med J. 2013;6(9):458-60. Epub 2013/10/18. doi: 10.4066/AMJ.2013.1806

20131806 [pii]. PubMed PMID: 24133538.

62. Panicker NK, Chandanwale SS, Sharma YK, Chaudhari US, Mehta GV. Chromoblastomycosis: Report of two cases on face from urban industrial area. Indian Dermatol Online J. 2013;4(4):371-3. Epub 2013/12/19. doi: 10.4103/2229-5178.120652

IDOJ-4-371 [pii]. PubMed PMID: 24350031.

63. Mandal RK, Banerjee S, Kumar P, Chakrabarti I. Non-healing verrucous plaque over upper limb for 1 year in a tea garden worker. Dermatol Online J. 2013;19(3):12. Epub 2013/04/05. PubMed PMID: 23552009.

64. Naha A, Rit K, Dey R. Chromoblastomycosis: A report of two cases from a tertiary care hospital of eastern India. Int J Health Allied Sci. 2013;2(3):200-2.

65. Ramraje SN, Gokhale J, Gupta S. Cutaneous chromoblastomycosis. J Case Reports. 2013;3(2):286-90.

66. Verma GK, Verma S, Singh G, Shanker V, Tegta GR, Minhas S, et al. A case of extensive chromoblastomycosis from North India. Braz J Microbiol. 2014;45(1):275-7. Epub 2014/06/21. doi: 10.1590/S1517-83822014005000025

bjm-45-275 [pii]. PubMed PMID: 24948945.

67. Shresta D, Kumar R, Durgapal P, Singh CA. Isolated nasal chromoblastomycosis. Indian J Pathol Microbiol. 2014;57(3):519-21. Epub 2014/08/15. doi: IndianJPatholMicrobiol_2014_57_3_519_138819 [pii]

10.4103/0377-4929.138819. PubMed PMID: 25118775.

68. Mittal A, Agarwal N, Gupta LK, Khare AK. Chromoblastomycosis from a Non-endemic Area and Response to Itraconazole. Indian J Dermatol. 2014;59(6):606-8. Epub 2014/12/09. doi: 10.4103/0019-5154.143537

IJD-59-606 [pii]. PubMed PMID: 25484395.

69. Bobba S. Case Study: Chromoblastomycosis. J Trop Dis. 2014;2(4):143.

70. Nath R, Barua S, Barman J, Swargiary P, Borgohain M, Saikia L. Subcutaneous Mycosis Due to *Cladosporium cladosporioides* and *Bipolaris cynodontis* from Assam, North-East India and Review of Published Literature. Mycopathologia. 2015;180(5-6):379-87. Epub 2015/07/23. doi: 10.1007/s11046-015-9926-x

10.1007/s11046-015-9926-x [pii]. PubMed PMID: 26198088.

71. Saxena AK, Jain S, Ramesh V, Singh A, Capoor MR. Chromoblastomycosis: demonstration of abundant microorganisms on microscopy of a scaly crust following intralesional corticosteroids. J Eur Acad Dermatol Venereol. 2015;29(1):189-90. Epub 2014/03/29. doi: 10.1111/jdv.12389. PubMed PMID: 24673685.

72. Dashatwar D, Kar S, Gangane N, Pol V, Madke B, Kulkarni S, et al. Chromoblastomycosis in a resident of a leprosarium. Lepr Rev. 2015;86(1):102-7. Epub 2015/06/13. PubMed PMID: 26065153.

73. Bajpai T, Nandedkar S, Bhatambare G, Gagrani N. Cutaneous chromoblastomycosis: A case report from central India. Int Med J Sifa Univ. 2015;2(3):59-61.

74. Bhramaramba K, Krishna L, Malini P, Srujana S, Shrvankumar O. A case of chromoblastomycosis in immunocompromised patient. MRIMS J Health Sci. 2015;3(1):79-80.

75. Mohammed S, Khan M, Kasetty HR. A rare case of oral chromoblastomycosis in India. J Evol Med Dental Sci. 2015;4(27):4737-41.

76. Roy P, Prasanna S, Laxmikant DV, Chaudhari CN. Chromoblastomycosis caused by *Cladophialophora carrionii* in a skin graft recipient. Med J Armed Forces India. 2015;74(4):389-92.

77. Khan S, Kumar A, Vinod V, Prabhakar V, Eapen M, Thomas J, et al. Chromoblastomycosis due to *Fonsecaea pedrosoi*: an old wine in a rare bottle. J Infect Dev Ctries. 2015;9(3):325-9. Epub 2015/03/17. PubMed PMID: 25771474.

78. Verma P, Karmakar S, Pandhi D, Singal A, Yadav P, Khare S. Chromoblastomycosis Caused by *Cladophialophora bantiana* in a Renal Transplant Recipient From Delhi, India. Skinmed. 2015;13(3):251-4. Epub 2015/09/19. PubMed PMID: 26380516.

79. Tanveer N, Mishra K. Chromoblastomycosis-A report of a rare fungal infection from non endemic region of north India. Indian J Basic Applied Med Res. 2015;4(2):72-5.

80. Raj HJ, Majumdar B, Jain A, Maiti PK, Chatterjee G. A Clinico-Mycological Study on Suspected Cases of Chromoblastomycosis: Challenges in Diagnosis and Management. J Clin Diagn Res. 2015;9(12):WC01-4. Epub 2016/01/28. doi: 10.7860/JCDR/2015/16199.6963. PubMed PMID: 26816977.

81. Bordoloi P, Nath R, Borgohain M, Huda MM, Barua S, Dutta D, et al. Subcutaneous mycoses: an aetiological study of 15 cases in a tertiary care hospital at Dibrugarh, Assam, northeast India. Mycopathologia. 2015;179(5-6):425-35. Epub 2015/01/19. doi: 10.1007/s11046-015-9861-x. PubMed PMID: 25596859.

82. Anjaneyan G, Jagadeesan S, Thomas J. Cytodiagnostic copper pennies in chromoblastomycosis. Indian Dermatol Online J. 2016;7(2):145-6. Epub 2016/04/09. doi: 10.4103/2229-5178.178085

IDOJ-7-145 [pii]. PubMed PMID: 27057510.

83. Bhat RM, Monteiro RC, Bala N, Dandakeri S, Martis J, Kamath GH, et al. Subcutaneous mycoses in coastal Karnataka in south India. Int J Dermatol. 2016;55(1):70-8. Epub 2015/08/13. doi: 10.1111/ijd.12943. PubMed PMID: 26267755.

84. Khan K, Mondal K, Dutta R, Mandal PK, Mandal R, Guha MM. Unusual Presentation of Cutaneous Chromoblastomycosis. Am J Dermatopathol. 2016. Epub 2016/05/06. doi: 10.1097/DAD.0000000000000612. PubMed PMID: 27149336.

85. Chauhan S, Kashyap N, Sood A, Jaryal SC, Thakur K. Microbiological profile of subcutaneous mycosis in a tertiary care hospital. Asian J Sci Tech. 2016;7(2):2311-2.

86. Krishna S, Shenoy MM, Pinto M, Saxena V. Two cases of axillary chromoblastomycosis. Indian J Dermatol Venereol Leprol. 2016;82(4):455-6. Epub 2016/06/10. doi: ijdvl_2016_82_4_455_174415 [pii]

10.4103/0378-6323.174415. PubMed PMID: 27279319.
